# Contested narratives: a qualitative analysis of abortion testimonies in Louisiana legislature

**DOI:** 10.3389/fgwh.2025.1533813

**Published:** 2025-04-17

**Authors:** Martha Silva, Jeni Stolow, Micki Burdick, Amy Mercieca

**Affiliations:** ^1^ Department of International Health and Sustainable Development, Celia Scott Weatherhead School of Public Health and Tropical Medicine, Tulane University, New Orleans, LA, United States; ^2^ Department of Social, Behavioral and Population Sciences, Celia Scott Weatherhead School of Public Health and Tropical Medicine, Tulane University, New Orleans, LA, United States; ^3^ Department of Women and Gender Studies, University of Delaware, Newark, DE, United States

**Keywords:** abortion, discourse, sexual violence, legislation, reproductive health

## Abstract

**Introduction:**

Following the Supreme Court's 2022 decision in *Dobbs v. Jackson Women's Health Organization*, Louisiana enacted a “trigger law” banning nearly all abortions. Attempts to reform existing restrictive legislation so as to allow for abortions under exceptions have been unsuccessful to date. This study aims to describe how abortion discourse is framed in public testimony around House Bill 346 in the 2023 Louisiana legislative session, which attempted to pass an abortion exception for pregnancy in the case of rape or incest.

**Methods:**

We conducted a conventional qualitative content analysis utilizing a rhetorical lens, using testimony transcripts from the May 10, 2023, Louisiana Administration of Criminal Justice Committee hearing. An iterative coding approach allowed us to categorize salient themes, language patterns, speaker characteristics, emotional tones, and rhetorical strategies. Demographic characteristics were ascribed to speakers based on perceived gender and race when not self-identified.

**Results:**

Testimony analysis revealed four primary themes: (1) conflicting representations of abortion, (2) religion's role in shaping discourse, (3) humanization of fetuses vs. pregnant individuals, and (4) debate over available resources for survivors and children. Abortion is represented as being traumatic, adding to the trauma caused by sexual violence, while representing childbearing as healing from trauma. Being conceived as a result of sexual violence is used as an identity marker worthy of protection. Religious rhetoric permeates testimony both in support and in opposition to abortion exceptions, making a “pro-life” stance the starting point for debate. Lastly, we find evidence of dehumanization of survivors' and others' experience.

**Conclusions:**

The testimonies around HB346 expose deeply polarized discourse that reflects moral, religious, and ethical conflicts, as well as mismatched conversations that are unlikely to persuade opposing sides. Addressing these dissonant narratives requires nuanced advocacy strategies and resources to support effective testimony.

## 1 Introduction

On June 24, 2022, the United States of America (USA) Supreme Court issued its ruling in Dobbs v. Jackson Women's Health Organization, overturning Roe v. Wade (1973), thereby eliminating Americans' constitutional right to abortion (1). Currently, abortion services are inaccessible to close to 30% of women in the country due to barriers related to legal and regulatory frameworks as well as broader social and economic inequities, despite being an integral part of sexual and reproductive health and rights (2). Even before the 2022 ruling, the USA had increasingly seen state-by-state actions to pass legislation that restricts abortion access, as well as “trigger laws”, laws that become enforceable after the occurrence of a specific event, that would immediately ban abortions in the case that constitutional protections were overturned. Louisiana is one of thirteen states that enacted a trigger law, originally enacted in 2006, that immediately banned abortion access on June 24, 2022, with the only exception to save the life of the pregnant woman (3).

In Louisiana, between July 1, 2022 and January 1, 2024, an estimated 34,525 people were survivors of completed vaginal rape, and a further estimated 4,287 of these survivors fell pregnant as a result of this rape (4). Public opinion polls indicate that Louisiana residents support exceptions to the ban on abortion in the case of rape and incest. In a 2023 Louisiana survey, 77% of respondents said a woman should be able to obtain a legal abortion if she becomes pregnant because of rape (5). Despite evidence of broad statewide support for this exception to the abortion ban, attempts to pass bills allowing these exceptions have failed in the 2023 and 2024 state legislative sessions.

Louisiana has been governed by conservative ideals with an anti-abortion governor from the Democratic party from 2016 to 2024 (6). Currently the Republican party controls the executive branch and both chambers of the Louisiana Legislature. Furthermore, Louisiana has become the first state to classify medication abortion drugs mifepristone and misoprostol as controlled substances, meaning they are treated similarly to opioids and other addictive and dangerous substances, imposing unnecessary barriers to accessing this medication even for non-abortion related care (7, 8). Louisiana is also an extremely hostile environment for reproductive rights and justice advocates and organizations who work in low resource environments. In this moment of extreme restrictions on sexual and reproductive health and rights, it is crucial to understand and deconstruct public discourse surrounding abortion, as well as the tactics used by policymakers to protect or restrict access to abortion services.

Through this study, we aim to understand social discourse surrounding abortion using publicly available Louisiana state legislative session testimony. This paper explores how abortion discourse is framed in public debate and testimony around House Bill 346 (HB346) in the 2023 Louisiana legislative session, which attempted to pass an abortion exception for pregnancy in the case of rape or incest. We include testimony from those in support of the exception (i.e., abortion should be accessible in the case of pregnancy caused by rape or incest), as well as those in opposition to the exception (i.e., abortion should not be accessible in the case of pregnancy caused by rape or incest).

## 2 Materials and methods

### 2.1 Design and population

A conventional content analysis utilizing a rhetorical lens was used to address the research question. Using this lens, we approach language as not just being an important part of any analysis of a situation; language is, instead, the way that meaning is created, sustained, and changed (9). Therefore, taking a discursive lens to these testimonies allowed us to understand how people make meaning out of their experiences, laws, and religion, among other things, through language. This discursive lens was used in the iterative design of the coding frame as well as the thematic analysis.

To complete this analysis, we utilized a publicly available video of the May 10th, 2023, hearing in the Louisiana Administration of Criminal Justice Committee. During this session, the HB346 bill sponsor presented the bill, members of the public provided testimony or submitted a witness card in support or opposition but choosing not to speak, and various representatives posed questions and offered comments. This was the only public hearing where this bill was heard. Although many opportunities for interactions between legislative representatives exist prior to the start of the legislative session, in addition to lobbying efforts from grassroots organizations and constituents, no other instances of debate were publicly available to use in this study. The video footage of this hearing was retrieved from the Louisiana House of Representatives Online Video Archive (https://house.louisiana.gov/h_video/VideoArchivePlayer?v=house/2023/may/0510_23_CJ). HB346 was one of several bills debated on that day. We analyzed arguments from all individuals participating specifically in the HB346 hearing (112 min).

### 2.2 Positionality statement

MS is a mixed-race, female-identifying Mexican immigrant with reproductive capacity, researcher and public health practitioner. JS is a white, female-identifying, researcher and public health practitioner. MB is a white, gender non-conforming communication and feminist scholar. AM is a white, female-identifying Louisiana native and long-standing reproductive rights legislative advocate. All four authors work within reproductive justice and believe abortion should be safe, legal, and accessible for those wanting to terminate pregnancies. Three of four authors reside in Louisiana.

### 2.3 Procedure

We extracted and reduced the full legislative video to include only the chosen bill (HB346). Happy Scribe, a virtual transcription service, was used to generate a transcript. A paid research intern checked the transcript for accuracy by viewing and listening to the video and reviewing the generated transcript, after which it was imported into NVivo version 14.

All four authors completed a full read of the transcript and viewed the video of the bill's hearing. Once reviewed, the research team iteratively, and collaboratively created the codebook. Codes were first independently identified using inductive coding, noting whether they came from reading the transcript or viewing the video. The codebook was then inductively constructed by comparing, debating, and synthesizing our identified codes. Subsequently, we independently coded the transcript, implementing a constant comparison approach, where we reviewed coding line by line discussing differences in interpretation. All four authors met to discuss coding throughout the coding process and conducted consensus coding after individual coding. Codes were then synthesized into categories such as speaker characteristics, various names for concepts (e.g., fetus, baby, victim, perpetrator) as well as perceived emotions, tone, and rhetorical strategies interpreted by the authors.

Lastly, we compiled descriptive statistics regarding participant characteristics. We ascribed demographic characteristics of the individuals testifying if the individuals did not describe themselves or their demographic characteristics (e.g., gender, race) in their testimony. We limited gender ascription to only two genders (male, female).

### 2.4 Study ethics

The Institutional Review Board at Tulane University determined that this study did not meet the criteria for human subjects research. All testimony analyzed is part of the public record and is directly downloadable through the Louisiana legislature webpage. Nonetheless, we do not directly identify any individual who provided testimony.

## 3 Results

Analysis includes testimony from 29 individuals, of which 21 were constituents and 8 were Louisiana representatives. Table 1 summarizes researcher-perceived characteristics of constituents and representatives testifying in support or in opposition of HB346. Twelve out of the 18 female speakers testified in support of the bill, while 3 of the 11 male speakers testified in support of the bill. Furthermore, the majority of the White individuals who testified did so in opposition, while the majority of the Black individuals who testified did so in support of the bill. As speakers began their testimony, all but two introduced themselves with a professional title, institutional or other role relevant to the hearing. Ten speakers were current or former public servants (i.e., former representative or former sitting judge), 6 of those in support and 4 in opposition. Four medical providers (Pediatricians and Obstetrics/Gynecologists) testified, 3 in support of the bill and 1 in opposition. Conversely, four religious leaders testified, 1 in support and 3 in opposition. Two people identified themselves as having been conceived by rape, 1 each in support and opposition; while 5 sexual assault survivors also disclosed their experience, 4 in support and 1 in opposition of the bill. Four other speakers identified themselves as employees of organizations known for advocacy in favor of legal access to abortion (i.e., Planned Parenthood Gulf Coast) or advocacy against access to legal abortion (i.e., Louisiana Right to Life). Nine people identified themselves as Christian or affiliated with a Christian institution, 2 in support and 7 in opposition.

**Table 1:** Sample characteristics.

Variable		# of Speakers in support (*n* = 15)	# of Speakers in opposition (*n* = 14)
Participant type	Constituents	9	12
	Representatives	6	2
Gender	Female	12	6
	Male	3	8
Race	White	6	12
	Black	8	2
	Other	1	0
Occupation or role in hearing (self-described)^a^	Current government figure	6	2
	Former government figure	0	2
	Medical provider	3	1
	Religious leaders	1	3
	Person conceived from rape/incest	1	1
	Sexual assault survivor/advocate	4	3
	Pro/anti choice advocate	1	3
	None mentioned	0	2
Religion	Christian	2	7
	Unknown/not identified	13	7

^a^ Not mutually exclusive.

We found four notable themes within our analysis, when taking into account both those in favor and in opposition of the bill: (1) various representations of abortion; (2) the role of religion in testimony discourse; (3) the humanization of the fetus with a simultaneous dehumanization of the gestating person; and (4) opposing views on available resources for birthing people. These themes allow us to document common patterns in testifying strategies for and against rape and incest exception bills as well as their consequences.

### 3.1 Representations of abortion

Various representations of abortion were apparent in the data across both support and opposition testimonies. Commonly among those in support of the bill, abortion was represented as “not an easy topic”, a part of healthcare, and an opportunity for healing after sexual trauma. Only those testifying in support of abortion exceptions represented abortion as healthcare. One abortion advocate and a medical doctor made it clear to include abortion as part of their definition of essential health care.

“We hear directly from patients who are harmed by the devastating laws banning essential health care [abortion] in our state… Louisiana must protect the most vulnerable among us, and that means doing everything in our power to ensure survivors of sexual assault can access the basic health care they need, including abortion if they choose it.” (Female, Black, in support)

Within the context of a pregnancy caused by rape; abortion was commonly represented as an opportunity for the survivor to heal after sexual assault trauma. Those providing testimony in support of the bill framed this type of pregnancy as a constant reminder of the trauma survived, and especially highlighted forced pregnancy and parenting as a particularly insidious form of trauma after experiencing sexual violence.

“Please do not put another young girl through the added trauma of being forced to parent on top of the already horrific act of rape or incest.” (Female, White, in support)

This narrative contrasts with the testimony put forward by individuals opposing the bill, describing parenthood of a child conceived by rape as a healing experience.

“There's a common argument that a child conceived by such a vile act will be a constant reminder of their pain. On the contrary, the innocence of the child often has a healing effect.” (Female, White, in opposition)

Furthermore, these speakers posit that allowing abortions in the case of pregnancies conceived by rape or incest puts into question the value of people conceived by rape or incest.

“Guys, yes, I may not have been conceived willingly. Yes, my mom may have been raped… but she has still been able to unconditional love me in a way that I have never felt loved by anyone else.. So, guys, don't question my value just because I wasn't conceived willingly. I can bring healing and hope to the world as well.” (Male, Black, conceived through rape, in opposition)

In a similar vein, speakers in opposition spoke of the need to support those conceived via sexual violence, equating mode of conception as a protected class similar to race, gender or ability status. Here we saw that this social justice framing was used across examples opposing abortion, such as: abortions trauma, abortion as a death sentence, abortion as a procedure that denies the value of people conceived by rape or incest, and abortion as a conspiracy tactic.

“I want to raise my children in a world where we do not discriminate based upon race, gender, disability, or way of conception” (Female, White, trauma survivor, in opposition)

The representation of abortion as a trauma was put forward by those in opposition to the bill, who believed that having an abortion would cause more harm to a person recovering from sexual violence, due to “*the guilt and turmoil of having her child killed”* (Female, White, in opposition) and does not undo the damage to the sexual assault survivor.

“Abortion is anti-woman, and to try to treat a woman with a trauma is not the right route” (Female, White, trauma survivor, in opposition)

For the fetus, abortion is represented as a death sentence for a “human being” who is also a subject to rights and due process under the law.

“No human being should be put to death without the due process of the law” (Male, White, in opposition)

Lastly, abortion was represented as being a part of a conspiracy, with one person testifying in opposition to the bill referring to an “abortion cartel” tied to the state government, using multiple references to criminality.

“Governor John Bell Edwards, the abortion industry and the media are trying to undermine the laws in our state regarding the protection of innocent babies in the womb and have done everything they can to hide the criminal activity of the abortion cartel. Now they prey on vulnerable women to sway support for their gruesome carnage by highlighting tragic circumstances to sway public support.” (Male, White, in opposition)

### 3.2 Role of religion

The use of religious discourse was prevalent throughout the hearing. The data indicate that both those in opposition as well as those in support of the proposed bill utilized religious discourse within the framework of Christianity. Among those in opposition to the bill, religious rhetoric was used to argue for choice as a false paradigm regarding pregnancy as well as the supposed inherent value of “life” in the womb. Among those in support of the bill, religious discourse was used in calls for freewill, as well as attempting to reframe the term “pro-life” in the argument.

Within the data, the use of religion or religious discourse was predominantly used in justifications for lack of choices. For example, those in opposition to the bill argued that pregnancy, even within the context of rape or incest, is always valued because it is and should always be God's choice to have a child put into the world.

“The Lord gave life to you and your parents chose to give you life and you are here on purpose and for purpose.” (Female, Black, rape survivor, in opposition).

Therefore, according to speakers in opposition, it is not a woman's choice to have an abortion, but for God to allow a child to come into the world.

The data also showed the importance thematically of those against the bill using religious discourse in arguments for personhood and the value of life:

“As a Christian, in particular, I take to heart the words of Jesus Christ when he said, ‘what you do or allow to be done to the very least of these, you do to me'. I cannot think of any segment of our society that is more defenseless or more vulnerable than the unborn.” (Male, White, in opposition).

Speakers in opposition to the bill used the Bible or cultural aspects of Christianity as justification for why abortion should be condemned in this case.

The data also showed that those who we identified as Christian, especially those who spoke in opposition to the bill, were often seemingly given the benefit of not having to bear the burden of proof in front of the conservative legislature. For example, it is mentioned that *“Louisiana is known as a state that protects the unborn.” (Male, in opposition*). Based on the makeup of the legislative body who ended up voting against this bill *(“Five yes, 10 nays”)* this is an accurate representation, not of the population, but of the local and state government itself.

As a result, both speakers in support as well as those in opposition to the bill used religious discourse and mentioned their proximity to religious organizations.

“And if we want to bring God into this, then we need to not only acknowledge that God gives us free will, but that he also knows the choices that we will make.” (Female, White, in support)

Speakers, such as the one above, brought God into the conversation, even if they were not associated with a religious organization. Many of these speakers also used the language of *“pro-life”* even if they were advocating for the passing of the bill. For example, speakers in support of the bill used language such as:

“If we are pro-life, we have to be concerned with more than just the baby in utero” (Black Female, conceived in rape, in support)

Or

“And when we think about valuing life, we can't sit here and say we truly value life if we don't value the life of the survivor of sexual assault who undoubtedly may consider, attempt, and succeed at committing suicide.” (Female, White, in support)

Lastly, a few speakers in support of the bill spoke out against this use of religious discourse throughout their testimonies. However, statements such as the one below from those in support of the bill did not occur often in response to the religious discourse used by those in opposition:

“We are supposed to separate church from state. We're supposed to understand that people have the freedom to choose whatever religion they want. By enforcing this, we take that right away from them. This bill does not mandate abortion. It just gives the option of that child, that woman that experienced that rape, may still decide to go through with the pregnancy.” (Representative, female, Black, conceived in rape, in support)

### 3.3 Humanization

The data showed a stark difference in how speakers humanized pregnant persons compared to fetuses and others. In this theme, “humanize” refers both to a person allocating personhood to someone/something as well as extending priority to a certain entity (such as a fetus). There were consistent differences in (1) if fetuses were humanized (i.e., called children, spoken about as a person) as well as (2) if that fetus was then given priority over the pregnant person. This debate is best captured from the exchange below:

**[REPRESENTATIVE]:** “I don't want to get into the back and forth because you and I would never agree. But I think we can agree on the fact that there are certain rights and liberties that are extended to all of us. And we are in a position now and today of talking about taking rights away from certain people within our population, particularly women. I'm trying to have a rationale in my mind, how do you, as someone who served on the battlefield as I did, think that's appropriate?” (Male, Black, voted in support)

**[RESPONSE]: “**Well, because the Constitution was based on the Declaration of Independence, which is based on natural law that listed among the inalienable rights, first and foremost, life, and then liberty. So if we can't respect the life [of a fetus], then the idea of liberty has no more meaning. So I was fighting for the Constitution in the same way, but I just had a priority of life that we should be defending.” (Male, White, medical provider, in opposition)

As mentioned previously in the section describing representations of abortion, the humanization of the fetus commonly led to the justification that if a fetus is a human, then it deserves to be treated as a person with rights.

“In my opinion, to be pro-life is to honor and protect the individual humanity and dignity of the unborn beginning at conception and to grant that individual the right to life and freedom, no matter the circumstances and challenges surrounding the pregnancy.” (Female, White, in opposition)

“[Abortion] is still a crime against nature as well as an unconstitutional robbing of an American's right to life, liberty, and the pursuit of happiness.” (Male, White, in opposition)

When humanizing language was focused on victims of rape or incest, speakers often discussed how important the concept of “choice” was to preserve a person's humanity. In these circumstances, abortion services were portrayed as a way to do so:

“I'm asking you to look within to imagine your loved ones, especially your children, in this position and to ask yourself, what would you want for them? Surely it is freedom. No matter how you have voted in the past, today you can do the right thing by your constituents and all Louisianans by supporting this bill.” (Female, Black, in support)

Humanizing language was deeply intertwined in dialogues around choice. The power of choice, choice of conception, and choice to testify were all key patterns within this testimony hearing. Often at the core of the testimony hearing was the debate about if the power of deciding choice should be at the state or the individual level.

“When I fought, and I'm not sure what branch you fought in, but when I fought, I fought for the Constitution. I fought on behalf of everyone having decisions to make of their own, not for the state to make.” (Male, Black, voted in support)

Further, the role of choice was seen as crucial to the debate as it relates not only to the choice of terminating a pregnancy, but also if the choice was made in the conception of that pregnancy**.**

“We talk a lot about choice when we talk about abortion on both sides, and I often hear if a woman chooses to have sex, but what if you don't choose? What happens when your choice is taken from you forcibly?” (Female, White, in support)

“To take their ability to make a decision about their bodies and their bodies, and that ability has already been taken, it will just continue to perpetuate the abuse to them.” (Female, Black, medical practitioner, in support)

Additionally, victims spoke of the strong necessity of their testimony to contextualize their experiences and provide evidence in favor of passing this bill. Due to the perceived severe necessity for this bill to pass in Louisiana, victims felt compelled to disclose their trauma, but felt the need to humanize the trauma discussed in the bill.

“This is a part of my story that I would rather have kept private, and I most likely would have if the stakes weren't so high for future survivors. Because our state has taken the choice away from survivors of rape and incest for the chance to recover, I sat my two boys down this past week so that they could hear the rest of the story, the part I never wanted public, and the part that I was not ready to share with them, from me.” (Female, White, in support)

Lastly, we found two examples of dehumanizing, not the fetus or pregnant person, but others. The intent of this dehumanizing was to create empathy for survivors of sexual violence seeking to terminate a pregnancy. The quote below illustrates both elements: using the image of an immigrant as a dangerous sexual predator as well as evoking the image of a known close person as the potential victim worthy of empathy.

“Our former President, Donald Trump, talks a lot about rapists, most of whom [are] from Mexico. And if that is our big bad monster in a scenario where some of our individuals may say, No exception. I would ask my colleagues, if the big bad monster raped your daughter, your niece, your granddaughter, your neighbor, your colleague, how would you feel?” (Representative, male, Black, in support)

### 3.4 Resources and support

Constituent testimony expressed the urgent need to support survivors of sexual violence, those with unwanted pregnancies, pregnant people and the unborn and born children. Those testifying in support of the bill expressed wishes for resources that focus on helping crime victims or the pregnant people access the care they need and mentioned specific resources for crime victims that have minimal barriers to access.

“I can think of six women in our… The hundreds and hundreds and hundreds of survivors we've organized over the past three years who reported to police. The vast majority do not. In Louisiana, to receive financial reimbursement from the Crime Victims Reparations Fund to get things like therapy, medical bills, lost wages, funerals paid for, for rape survivors, you haven't had to report to police to receive those services for a long time. If you get a forensic medical exam, that automatically gets transmitted to the Crime Victims Fund, and then that is your proof, essentially, and then you can receive those benefits from the state.” (Female, White, in support)

Other testimony stressed the significance of not tying police reporting to access of funds. Constituents described the aftermath of a sexual assault, and particularly the interaction with police and the legal system, as extremely retraumatizing.

“Yeah, I mean, the re-traumatization that we hear about from our members in Louisiana Survivors for a Forum ranges from their friends and family not believing them, to interactions they've had with police and the criminal legal system that was retraumatizing, and absolutely not being able to have access to the medical care you need following an experience of rape or incest is re-traumatization, absolutely.” (Female, White, in support)

Constituents testifying in support of the bill described a lack of resources to support children once they are born, and a lack of resources for family mental health.

“I want to say this as well, because of the mental health crisis and the lack of resources and access to mental health care, that adds on to the complexity of the situation because mental health services are really very limited in the state, definitely as it relates to children.” (Female, Black, in support)

In contrast, those in opposition used discourses of abundance when discussing resources for pregnancy and in support of families, mentioning the availability and accessibility of significant resources up to a free college education for orphaned children.

“We provide many ministries. I hate to say this just on behalf of Louisiana Baptist because I know Catholics have tremendous ministries that do the same thing. Assemblies of God, Pentecostals, Methodists. There are just a number of churches that do likewise. But we provide health care and support and counseling for mothers and their unborn children. In some situations, we provide health care for the baby up to two years after birth. We provide adoption care, foster care, and orphan care. In fact, if you're an orphan in our ministry and you want to go to college, we give you a free college education. We take care of women who are victims of domestic abuse. We rescue women and children out of human trafficking. Those are some of the ministries. But I just want you to know we put our money where our mouths are on this issue.” (Male, White, in opposition)

Lastly, one person who testified in opposition to the bill alluded to the number of resources that would be needed to implement an exception to the abortion ban for pregnancies caused by rape and incest. We found the only allusion to false claims of rape during this bill hearing in this statement.

“The proposed law would guarantee clogged courtrooms and jails bursting at the seams from all of the false claims of rape when in fact the sexual encounter was consensual. All a woman would have to do is tell her doctor that she said no at some point in the sexual encounter, and the doctor would chalk it up as rape and the abortion would be performed…Old girlfriends will clamor for the opportunity to put their old boyfriend or ex-lover behind bars in order to dispense with the inconvenience of giving birth. Meanwhile, there is no way to truly know who is being falsely imprisoned. In theory, prostitutes could claim rape on a repeated basis simply to avoid losing downtime due to multiple pregnancies…This bill will simply reduce the present law to meaningless legislation as well as create a disastrous legal nightmare in our courts and prisons paid for with taxpayers' dollars.” (Male, White, in opposition)

## 4 Discussion

The analysis of abortion-related rhetoric of HB 346 reveals a deeply polarizing debate with distinct representations of abortion aligned with support or opposition to the proposed bill. Supporters of the bill frame abortion as an essential aspect of healthcare and a critical option for survivors of sexual violence to heal, emphasizing the trauma of forced pregnancy and parenting. In contrast, opponents depict abortion as a form of trauma, a moral wrongdoing equivalent to a death sentence for a human being, and a social justice issue that devalues lives conceived through rape or incest. Religious discourse predominantly, but not exclusively, influences arguments against abortion, reinforcing themes of divine will and personhood of the fetus. Both sides humanize their perspectives—pro-bill advocates highlight the necessity of choice for preserving humanity, especially for survivors, while anti-bill speakers prioritize the fetus' right to life. Additionally, the debate extends to the availability and sufficiency of resources and support for survivors and children. Proponents of the bill stress the lack of adequate resources for mental health care, support for survivors, and child welfare, arguing that the current system fails to meet these critical needs. Conversely, opponents claim an abundance of resources provided by religious and community organizations, highlighting comprehensive support services for mothers and children, including healthcare, adoption, and educational opportunities. This dichotomy underscores the complex interplay of moral, religious, and ethical considerations in the legislative discourse on abortion.

Our findings confirm several themes identified in previous studies. Like Evans et al. we observed framing of both forced pregnancy as trauma and abortion as trauma. However, unlike other studies, we found few examples vilifying people who receive abortion care as lazy, selfish and immoral as well as those who provide or support them (10). Unlike Evans and colleagues, we saw limited discourse around disbelieving survivors of assault (11). With exception of one person providing testimony in opposition, most stayed away from antagonizing survivors, but separated the “tragic circumstances” of the pregnancy from the pregnancy outcome. Those in support of the bill focused on extremes, innocent children being forced to birth a child, likely because circumstances such as those are perceived as more likely to evoke empathy and support for an exception. As Evans points out, this plays into an expected “hierarchy” of abortion narratives, perpetuating ideas that the more horrific the rape story is, the more justifiable an abortion: “Legitimizing the personhood of fetus provides the emotional scaffolding for legislators to deem most abortions unnecessary, including in case of rape or incest. Right to life of a fetus is viewed as of equal if not greater importance than the well-being of a pregnant person” (11). Additionally, other studies have documented newer trends in public discourse, appropriating civil rights discourse to disguise abortion bans as an attempt to protect women, which we also find in this study (10, 12).

Lastly, like Evans et al, we see attempts to call for empathy and nonpartisanship from those in support, using hypothetical examples of close friends and relatives to evoke empathy (11). However, we also found, beyond this proximity used for empathetic purposes, an exclusionary aspect to these rhetorical choices. By using proximity to one's personal experiences as a rhetorical strategy to evoke empathy for the “other side,” many marginalized peoples become excluded or “othered” in the process. For example, within our theme of dehumanization, we found arguments from supporters that focused on evoking personal and individualized calls to care about the problem of pregnancy resulting from rape or incest. The rhetorical use of a statement such as “what if it was your wife/daughter/mother?” individualizes the problem while othering those who are, most likely, not in a privileged position. Within these themes and examples, there exists an unstated implication that what happens to people that are not proximal to you is less worthy of your consideration or empathy. These assumptions also flatten all experiences of women and pregnant people together, even when embodied experiences of trauma (in the case of rape) or healthcare (in the case of abortion) are not universal. Even the word “rape” itself is often a contested term in a discursive sense as it can mean a variety of things to different survivors, sometimes limiting agency as well as dichotomizing experiences (13). Therefore, flattening of complex experiences in these legislative conversations becomes a theme throughout.

Importantly the mismatch of testimony topics (i.e., forced parenthood is harmful vs. the fetus is not at fault for the crime that led to its conception) is leading to no progress in these sessions with no foreseeable common ground. Policy debates surrounding abortion legislation in many states are not guided by scientific evidence. When such evidence is used, it is most often framed to support pre-existing ideological posture (14). Instead of evidence-based arguments, morality frames are often used in legislative debates by both politicians on the support and opposition of abortion access. For those in support of abortion access, the most frequently used morality frames are that of women's rights, pragmatic consequences of lack of access to legal abortion, individual and state (i.e., intrusion and over-regulation by the state), and social justice. For those in opposition to abortion access, fetal personhood is a commonly used morality frame, as is women's safety rather than autonomy (15).

The framing of abortion opposition has evolved in the USA. Historically, religious rhetoric was widely used in public discourse to argue against access to abortion services (16). For example, religious rhetoric has been used by Republicans under the guise of a call for “family values,” to rally efforts against abortion under any circumstances (16, 17). However, recent qualitative policy analyses have found a rise in the use of scientific misinformation strategies and a lack of religious rhetoric. This evidence was found in passed legislation such as a 6-week abortion bans in Georgia and South Carolina, as well as fetal heartbeat bills. These studies documented the use of misinformation via scientific framing such as the oversimplification of complex concepts, the use of incorrect scientific facts, as well as framing science withing value and morality lenses (10, 12). By not addressing the mis/disinformation presented by those in opposition, reproductive rights advocates are missing the opportunity to highlight the gaps in the falsified statements. For example, there is a recognized unmet need for mental health services in Louisiana, despite the rhetoric of abundance utilized by those in opposition to the bill (18). Furthermore, the two mismatched conversations between those in support and in opposition are further creating echo chambers where those in support of access expanding bills are only producing evidence they see as compelling, with those in opposition doing the same. It is not reasonable to expect these echo chambers to dissolve and both sides present the same evidence for the same arguments. It is time for reproductive advocates to go on the offense and address the opposition's echo-chamber in order to ensure access to safe and legal abortion services.

Lastly, more concerted efforts are needed to expand and coordinate with advocacy beyond the legislative system, as evidence indicates that abortion exceptions do not successfully enable access to abortion services for pregnant people who fall into these categories, forcing people to either carry a pregnancy to term or travel to access healthcare (4, 19).

### 4.1 Implications and recommendations

Results presented in this paper summarize main themes and use of language pertaining to abortion, specifically for survivors of sexual violence. These findings highlight the need for a better understanding of what effective strategies look like in legislative committee. Table 2 summarizes our recommendations for future testimony sessions. It is imperative to point out that these recommendations are not being made in a vacuum. We acknowledge the burnout, low resources, and lack of support that reproductive rights and reproductive justice organizations have. The following recommendations are made in tandem with a call for resource support for the overworked and under-assisted organizations, especially those in Louisiana.

**Table 2:** Recommendations for future legislative sessions.

Testimony strategy recommendation	Description
Identify the purpose of the testimony: persuasion vs. building a record of stories for awareness raising	Consider what the purpose of testimony is. Testimony for persuasion is unlikely to succeed. However, Testimony is able to become a way to build a record of stories that may not have been told otherwise. This record in and of itself, as well as people giving testimony on their own terms and consent, can be subversive as well as bring together testifiers to create and extend collective rhetoric that eventually sway and put pressure on both court and public opinion (20).
Collaborate across research, practice, and advocacy efforts	Connect to prioritize research needs, testimony strategy, and any resource gaps identified by reproductive rights organizations.
Review recordings and reactions to past testimony to inform upcoming sessions	Do not recycle previous testimony points/arguments. Take the defensive move to anticipate possible strategies by the opposition and prepare accordingly (e.g., anticipate opposition's use of social justice rhetoric)
Embed clear, consistent, compelling scientific evidence within testimony	While scientific information is not universally seen as persuasive (14), providing strong, consistent scientific evidence to the people providing testimony can create a cohesive narrative and strengthen arguments in favor of abortion access.
Address current trends in misinformation/disinformation	Proactively prepare statements to combat commonly mentioned mis/disinformation testimony heard in the court, local media, social media, and national media; for example, the unmet need for mental health services in Louisiana that stands in opposition to the rhetoric of abundance (18, 21).
Prepare religious evidence in support of your testimony	Work with religious leaders to compile and provide religious evidence to combat religious arguments against reproductive rights (22).
Support the use of personal narratives in testimony	Personal narratives can be compelling in testimony hearings but use cautiously as they may be retraumatizing to the witness. There exists a hierarchy of grief, anger, and compassion in the courtroom, with grief and compassion being more traditionally understood and accepted by the court or members of a judicial body than anger (23). Storytelling of experiences and emotions within courts are not neutral, but come with a set of historical, political, and cultural assumptions (24).
Leverage key actors in testimony hearings	Acknowledge who the most likely influencers are in the hearing: who represents the majority of legislators, religious leaders, healthcare providers, etc.

### 4.2 Limitations

There are several limitations to this study. First, we were unable to collect self-reported demographic information of legislators and community members giving testimony in public session debates. We ascribed speakers' demographic characteristics based on our team's perceptions of their race, gender and age. This analysis is also limited to one bill within a very particular geographical context. Analysis of a single bill hearing does not allow for an understanding of rhetorical evolution in this context. Future studies can build upon these findings to explore possible strategies for future hearings of these bills and others.

## 5 Conclusion

Policy debates and surrounding abortion legislation in many states, including Louisiana, are rife with complex uses of discursive strategies tied to political, moral, religious, and ethical histories and conflicts. This study found that those in opposition and those in support of abortion access are leading dissonant conversations within political spaces. We hypothesize that these disjointed debates are a driver of the lack of progress in expanding legal protections for abortion. Further, we recommend several strategies for addressing these disconnected debates. In hand with these recommendations, we recognize that nationally, and especially in Louisiana, reproductive rights organizations are working tremendous hours with limited personnel, material resources, legal resources, and financial resources. This study highlights the need for more research, partnerships, and documentation aimed at supporting reproductive rights advocates to better carry abortion access expanding bills through the current political context.

## Data Availability

A publicly available dataset was analyzed in this study. This data can be found here: https://house.louisiana.gov/h_video/VideoArchivePlayer?v=house/2023/may/0510_23_CJ
